# P-2020. Combination Therapy with Remdesivir and Corticosteroids is Associated with Lower Mortality Risk vs. Corticosteroids Monotherapy in Patients Hospitalised for COVID-19

**DOI:** 10.1093/ofid/ofae631.2177

**Published:** 2025-01-29

**Authors:** Essy Mozaffari, Aastha Chandak, Robert L Gottlieb, Chidinma Chima-Melton, Mark Berry, Thomas F Oppelt, Jason F Okulicz, Alpesh N Amin, Paul E Sax, Andre Kalil

**Affiliations:** Gilead Sciences, Foster, California; Certara, New York, New York; Baylor University Medical Center, Dallas, Texas; University of California Los Angeles, Los Angeles, California; Gilead Sciences, Inc., Foster City, California; Gilead Sciences, Inc, Foster City, California; Gilead Sciences, Inc., Foster City, California; University of California, Irvine, Orange, California; Brigham and Women’s Hospital; Harvard Medical School, Boston, MA; University of Nebraska Medical Center

## Abstract

**Background:**

Most clinical guidelines recommend treatment of patients hospitalized for COVID-19 with remdesivir (RDV), while adding a corticosteroid such as dexamethasone (DEX) is recommended among patients with hypoxemia. Since other corticosteroids (CORT) such as prednisone, prednisolone, methylprednisolone, and hydrocortisone can be used in place of dexamethasone or for other underlying conditions, we examined all-cause mortality in hospitalised COVID-19 patients initiating RDV+CORT vs. CORT monotherapy with information from the more recent COVID-19 era.

**Table 1:**
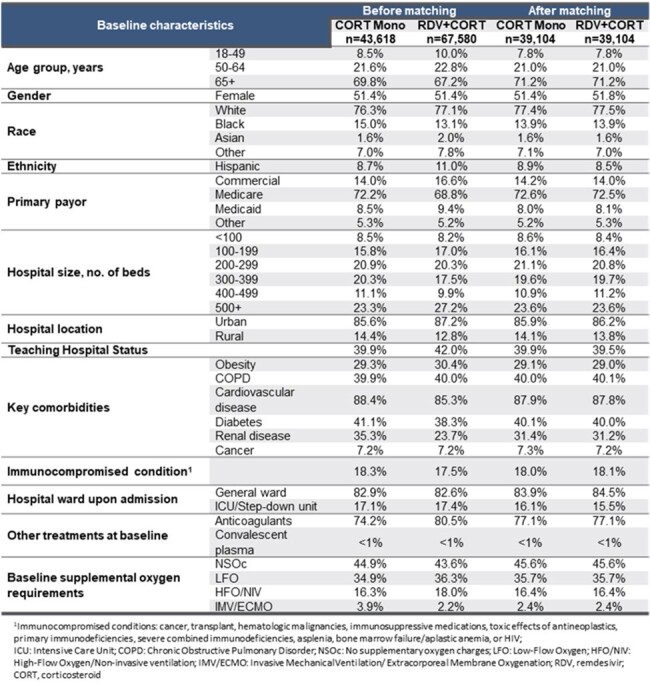
Baseline characteristics before and after matching

**Methods:**

Using the PINC AI Healthcare database, adults hospitalised during the Omicron period (December 2021 to April 2023) with a primary discharge diagnosis of COVID-19 and flagged as “present-on-admission” and initiating RDV+CORT or CORT monotherapy in the first 2 days of hospitalisation (baseline period) were included. Patients were matched using 1:1 preferential within-hospital propensity matching. Cox Proportional Hazards Model was used to examine time to 14- and 28-day mortality.

**Figure 1:**
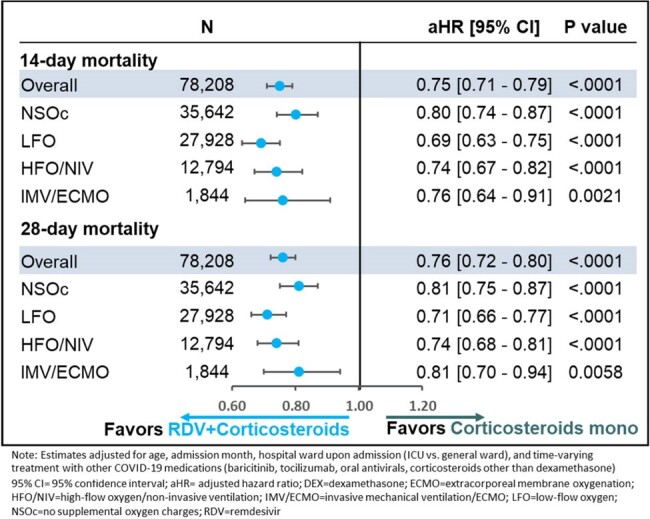
14- and 28-day mortality in patients hospitalized for COVID-19 receiving RDV+Corticosteroids or Corticosteroids monotherapy by supplemental oxygen requirements

**Results:**

Among 151,215 COVID-19 hospitalised patients, 67,580 (45%) initiated RDV+CORT and 43,618 (29%) CORT monotherapy (24% DEX monotherapy; 5% non-DEX corticosteroid monotherapy) in the first 2 days of hospitalization. Of the latter, RDV was not added for 90% (n=39,286) of patients after the first two days of hospitalization. A total of 39,104 RDV+CORT patients were matched 1:1 to CORT monotherapy patients. Post-matching balance was achieved with 71% age 65+, 36% LFO, 16% HFO/NIV, and 2% IMV/ECMO at baseline (Table 1).

After adjusting for baseline and clinical covariates, RDV+CORT had a significantly lower mortality risk compared to CORT monotherapy overall and across all supplemental oxygen requirements at 14 days and at 28 days (Figure 1). Similar results were obtained for RDV+DEX vs. DEX monotherapy (Figure 2).

**Figure 2:**
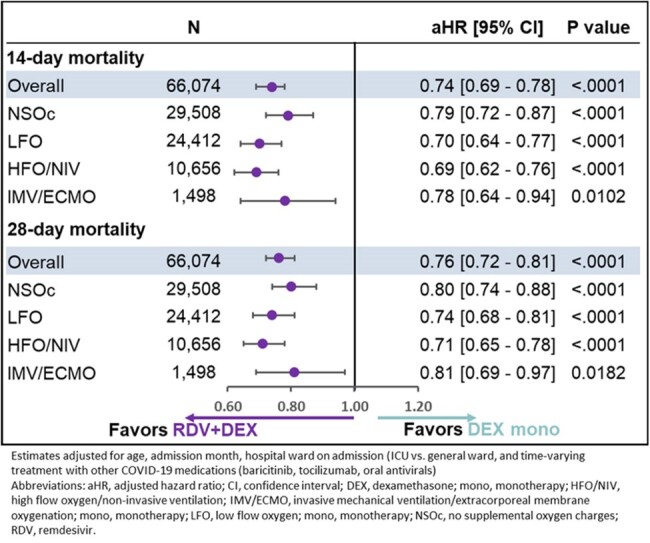
14- and 28-day mortality in patients hospitalized for COVID-19 receiving RDV+Dexamethasone vs. Dexamethasone monotherapy by supplemental oxygen requirements

**Conclusion:**

The study highlights the important role of antiviral therapy with remdesivir in improving clinical outcomes in patients hospitalized with COVID-19, supporting the guidelines that recommend its use. The data also expose non-adherence to these guidelines, as corticosteroid monotherapy should rarely be used, especially in those who do not require supplemental oxygen.

**Disclosures:**

Essy Mozaffari, PharmD, MPH, MBA, Gilead Sciences, Inc.: Employee|Gilead Sciences, Inc.: Stocks/Bonds (Public Company) Aastha Chandak, PhD, Gilead Sciences Inc.: My organization (Certara) was contracted by Gilead to conduct this study Robert L. Gottlieb, MD, AbbVie: Advisor/Consultant|AbCellera: Stocks/Bonds (Public Company)|AstraZeneca: Advisor/Consultant|Eli Lilly: Advisor/Consultant|Gilead Sciences Inc.: Advisor/Consultant|Gilead Sciences Inc.: Honoraria|Gilead Sciences Inc.: travel support, gift-in-kind to his institution to facilitate an unrelated academic-sponsored clinical trial NCT03383419|GSK Pharmaceuticals: Advisor/Consultant|Johnson & Johnson: Advisor/Consultant|Pfizer: Honoraria|Regeneron: Grants or contracts to institution|Roche: Advisor/Consultant|Roivant Sciences (Kinevant Sciences): Grants or contracts to institution Chidinma Chima-Melton, MD, Gilead Sciences: Advisor/Consultant Mark Berry, PhD, Gilead Sciences, Inc.: Employee|Gilead Sciences, Inc.: Stocks/Bonds (Public Company) Thomas F. Oppelt, PharmD, BCPS, Gilead Sciences, Inc: I am an employee of Gilead Sciences, Inc|Gilead Sciences, Inc: Stocks/Bonds (Public Company) Jason F. Okulicz, MD, Gilead Sciences Inc.: Employee|Gilead Sciences Inc.: Stocks/Bonds (Public Company) Alpesh N. Amin, MD, MBA, Alexion: Advisor/Consultant|Aseptiscope: Advisor/Consultant|AstraZeneca: Advisor/Consultant|Bayer: Advisor/Consultant|Dexcom: Advisor/Consultant|Eli Lilly: Advisor/Consultant|Ferring: Advisor/Consultant|Gilead: Advisor/Consultant|GSK: Advisor/Consultant|Heartrite: Advisor/Consultant|Nova Nordisk: Advisor/Consultant|Pfizer: Advisor/Consultant|Renibus: Advisor/Consultant|Reprieve: Advisor/Consultant|Salix: Advisor/Consultant|Seres: Advisor/Consultant|Spero: Advisor/Consultant

